# Integrated analysis of a ceRNA network reveals potential prognostic lncRNAs in gastric cancer

**DOI:** 10.1002/cam4.2760

**Published:** 2020-01-10

**Authors:** Mingran Qi, Bingxin Yu, Huiyuan Yu, Fan Li

**Affiliations:** ^1^ Department of Pathogenobiology The Key Laboratory of Zoonosis Chinese Ministry of Education College of Basic Medicine Jilin University Changchun Jilin China; ^2^ Department of Ultrasound China‐Japan Union Hospital of Jilin University Changchun Jilin China; ^3^ School of Public Health Jilin University Changchun Jilin China; ^4^ The Key Laboratory for Bionics Engineering Ministry of Education Jilin University China Changchun Jilin China; ^5^ Engineering Research Center for Medical Biomaterials of Jilin Province Jilin University Changchun Jilin China; ^6^ Key Laboratory for Biomedical Materials of Jilin Province Jilin University Changchun Jilin China; ^7^ State Key Laboratory of Pathogenesis, Prevention and Treatment of High Incidence Diseases in Central Asia Xinjiang China

**Keywords:** biomarkers, ceRNA network, gastric cancer, long noncoding RNA, risk score

## Abstract

Long noncoding RNAs (lncRNAs) have important biological functions as competing endogenous RNAs (ceRNAs) in tumors, yet the functions and regulatory mechanisms of lncRNA‐related ceRNAs in gastric cancer have not been fully elucidated. In this study, we constructed a lncRNA‐miRNA‐mRNA ceRNA network and identified potential lncRNA biomarkers in gastric cancer. Basing on the RNA profiles downloaded from The Cancer Genome Atlas (TCGA) platform, the gastric cancer‐specific differentially expressed lncRNAs, miRNAs, and mRNAs were screened for constructing a ceRNA network using bioinformatic tools. The enrichment analysis of the biological processes in Gene Ontology and the Kyoto Encyclopedia of Genes and Genomes pathways was performed on the ceRNA‐related DEmRNAs. According to the modularization of protein‐protein interaction (PPI) network, we extracted a ceRNA subnetwork and analyzed the correlation between the expression of the lncRNAs involved and specific clinical features of patients. Next, the expression of highly up‐regulated in liver cancer (HULC) and RP11‐314B1.2 showed significant changes in several pathological processes involved in gastric cancer, and nine lncRNAs were found to be correlated with the overall survival of patients with gastric cancer. Through the univariate and multivariate Cox regression analyses, two lncRNAs (LINC00106 and RP11‐999E24.3) were identified and utilized to establish a risk score model for assessing the prognosis of patients. The analysis results were also partially verified using quantitative real‐time PCR. The findings from this study indicate that HULC, RP11‐314B1.2, LINC00106, and RP11‐999E24.3 could be considered as potential therapeutic targets or prognostic biomarkers in gastric cancer, and provide a new perspective for cancer pathogenesis research.

## Graphical abstract

We constructed a lncRNA‐associated ceRNA network basing on the RNA profiles downloaded from The Cancer Genome Atlas (TCGA) platform, and performed integrated analysis of the ceRNA network to reveal potential prognostic lncRNAs in gastric cancer.

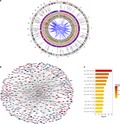

## 1 BACKGROUND

Gastric cancer is the fifth most common malignancy in the world.^1^ According to the most recent global cancer statistics, there will be more than 1 million new cases of gastric cancer in 2018, along with 783,000 attributed deaths, making it the third most deadly form of cancer worldwide.^2^ *Helicobacter pylori* (*H pylori*) infection, salt‐preserved foods and low fruit intake, tobacco smoking, and alcohol consumption are all risk factors for gastric cancer.^3^, ^4^ However, the development of gastric cancer is a complex and multifaceted process that results from the involvement of multiple molecules and biological mechanisms, and its pathogenesis remains largely unknown. Currently, there is a lack of effective biomarkers for the diagnosis and prognosis of gastric cancer.

Noncoding RNAs (ncRNAs), a class of RNAs with no known protein‐coding functions, can be categorized into three subclasses according to their size including, short with 20‐50 nucleotides (nt), mid with 50‐200 nt, and long with >200 nt.^5^, ^6^ MicroRNAs (miRNAs), a type of short ncRNAs with approximately 22 nt,^7^ can partially or entirely bind to the 3′‐untranslated regions (3′‐UTRs) of target genes to promote the degradation of targeted mRNAs and translation suppression, along with the negative regulation of gene expression at posttranscriptional levels.^8^, ^9^ Long noncoding RNAs (lncRNAs) have more than 200 nt and are known to participate in several physiological and pathological processes, including gene imprinting, chromatin modification, epigenetic regulation, dosage compensation, cell cycle regulation, and cell differentiation in terms of RNA.^10^, ^11^ Recently, lncRNAs were found to be involved in the occurrence and development of malignant tumors. For example, colon cancer‐associated transcript 1 (CCAT1), HOX transcript antisense RNA (HOTAIR), and plasmacytoma variant translocation 1 gene (PVT1) were all found to have essential roles in tumor growth and metastasis.^12^, ^13^, ^14^, ^15^ LncRNA functions through a variety of mechanisms. For example, the competing endogenous RNA (ceRNA) hypothesis presented by Salmena et al^16^ suggested that multiple RNAs can interact with each other through miRNA response elements (MREs). The ceRNA networks combine the function of protein‐coding mRNAs with that of ncRNAs such as circular RNAs (circRNAs), miRNAs, or lncRNAs.^17^ The physiological functions of cells may be disturbed when some RNAs in the ceRNA network are abnormally expressed as they can competitively affect the expression of other RNAs. This can have a profound impact on the occurrence and progression of many diseases, including cancer.

**Figure 1:**
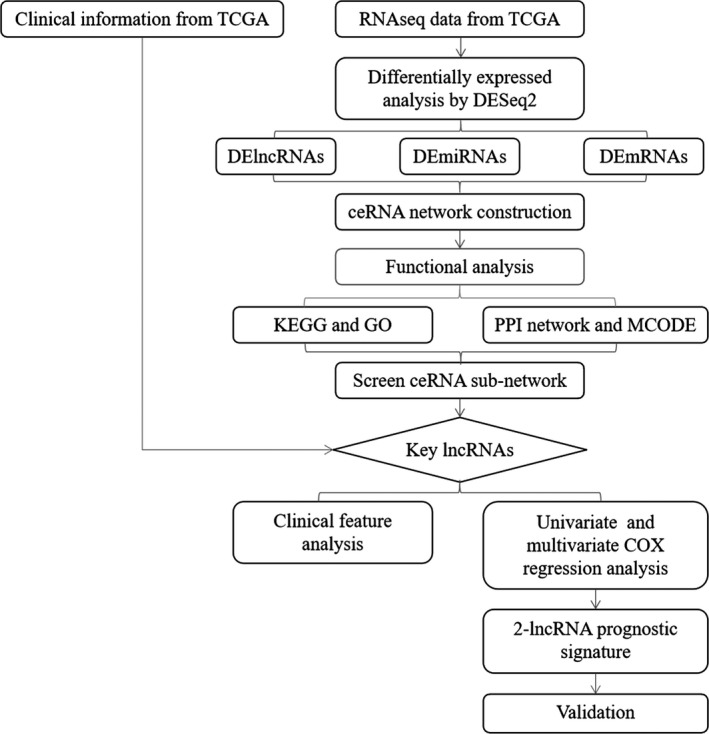
Flowchart of this research showing steps involved in construction of lncRNA‐based prognostic risk score model

In this study, gastric cancer data were collected from The Cancer Genome Atlas (TCGA), which included RNA expression patterns of 372 tumor samples and 32 nontumor samples. According to bioinformatic predictions and analyses, a ceRNA network with 207 lncRNAs, 73 miRNAs, and 224 mRNAs was constructed. Meanwhile, the potential biological functions and the clinical features of lncRNAs were also identified. The expression of specific lncRNAs was validated by quantitative real‐time PCR (qRT‐PCR) in gastric cancer cells. And, the flowchart of this research is shown in Figure 1. Through this analysis, we aim to understand the pathogenesis of gastric cancer better and reveal some potential lncRNA biomarkers.

## 2 Materials and methods

### 2.1 Data collection

The gastric cancer RNA expression profiles (level 3) were downloaded from TCGA (https://portal.gdc.cancer.gov/) database using the RTCGAToolbox R package.^18^ This included count data of RNA sequencing containing mRNAs and lncRNAs expression profiles via RNASeqV2 and microRNAs sequencing data analyzed by Illumina HiSeq 2000 miRNAseq platforms. A total of 404 samples were analyzed, which included 372 tumor tissue samples and 32 noncancerous tissue samples. Annotations from the GENCODE project (http://www.gencodegenes.org) were used to identify lncRNA expression data from mRNA expression profiles.^19^ Clinical and demographic data of 372 gastric cancer patients were extracted from the TCGA, including age, gender, race, pathological stage, pathological tumor (T), pathological node (N), pathological metastasis (M), and *H pylori* infection.

### 2.2 Screening of differentially expressed RNAs

The RNA expression profile data downloaded from the TCGA were analyzed with the DESeq2 R package.^20^ By setting the |log_2_ fold change|> 1 with an adjusted false discovery rate (FDR) of *P* < .001 as the criteria, the differentially expressed lncRNAs (DElncRNAs), differentially expressed mRNAs (DEmRNAs), and differentially expressed miRNAs (DEmiRNAs) were screened for subsequent analysis. The Circos plot was visualized using the RCircos R package.

### 2.3 Prediction of lncRNA‐miRNA and miRNA‐mRNA interactions

We used DElncRNAs, DEmiRNAs, and DEmRNAs to construct the regulatory network. First, we predicted the lncRNA‐miRNA interactions based on DEmiRNAs using the experimental module DIANA‐LncBase Version 2 (http://www.microrna.gr/LncBase).^21^ Secondly, TargetScan (http://www.targetscan.org),^22^ miRDB (http://www.mirdb.org/),^23^, ^24^ and DIANA‐TarBase Version 8 (http://www.microrna.gr/tarbase) 25 were used to predict the interactions between DEmiRNAs and DEmRNAs, and the miRNA‐mRNA interactions detected in these three databases were adopted. Ultimately, we retained interactions with the DElncRNAs, DEmiRNAs, and DEmRNAs, and constructed a lncRNA‐related ceRNA network with Cytoscape Version 3.6.1 based on how lncRNAs can affect the function of miRNAs and act as miRNA sponges to regulate mRNA expression.^16^

### 2.4 Gene Ontology and the Kyoto Encyclopedia of Genes and Genomes pathway enrichment analysis

The DAVID Version 6.8 (Database for Annotation, Visualization, and Integrated Discovery, http://david.abcc.ncifcrf.gov/) 26 and Metascape (http://metascape.org) 27 tools were used for the functional analysis of DEmRNAs in the ceRNA network. The biological processes in Gene Ontology (GO) and the Kyoto Encyclopedia of Genes and Genomes (KEGG) pathways (*P* < .05) were selected to analyze their biological function. The significant enrichment results were visualized using the ggplot2 R package 28 and Cytoscape Version 3.6.1.

### 2.5 Integration of protein‐protein interaction (PPI) networks and module analysis

To further investigate the function of DEmRNAs in the ceRNA network at the protein level, the Search Tool for the Retrieval of Interacting Genes (STRING) database was used.^29^ ceRNA network‐specific DEmRNAs were mapped to STRING, and the median confidence score (0.4) was used to evaluate the protein interactions. Next, Cytoscape Version 3.6.1 was used to construct the PPI network, and the Plug‐in Molecular Complex Detection (MCODE) was used to filter the module of the PPI network based on the criteria of having an MCODE score ≥3 and number of nodes ≥3. In addition, mRNAs in the modules were selected as core RNAs to constitute a subnetwork.

### 2.6 Clinical features analysis of key lncRNAs

The lncRNAs from the subnetwork were chosen as key lncRNAs to study their associations with specific clinical signatures of the patients, including age, gender, tumor stage, tumor infiltration, lymphatic and distant metastasis, and *H pylori* infection with the edgeR R package 30 and criteria of |log_2_ fold change|> 1 and an adjusted FDR of *P* < .05. In addition, the R packages of survival and survminer were used to analyze the correlation between the expression of key lncRNAs and overall survival (log‐rank test *P* < .5). Meanwhile, the univariate and multivariate Cox regression analyses were performed on the relative expression of key lncRNAs to construct a lncRNA‐associated risk score model, and evaluate whether the risk score model is an independent prognostic indicator. Receiver operating characteristic (ROC) analysis was performed with the pROC R package.^31^

### 2.7 Regression analysis of key lncRNAs and DEmRNAs

The regression analysis of the relative expression level of key lncRNAs and core DEmRNAs was analyzed and visualized by ggpubr and ggplot2 R packages by setting *r* ≥ .3 and *P* < .05 as the criteria.

### 2.8 RNA extraction and qRT‐PCR validation

The normal human gastric epithelial cell line, GES‐1, and human gastric cancer cell lines, BGC‐823, HGC‐27, MGC‐803, SGC‐7901, AGS, and MKN‐28, were maintained in our lab. The cells were cultured in Dulbecco's Modified Eagle's Medium (DMEM, Gibco, Gaithersburg, MD, USA) supplemented with 10% fetal bovine serum (FBS, Gibco, Gaithersburg, MD, USA) and antibiotics (penicillin 100 U/mL, streptomycin 100 µg/mL, Sigma Aldrich, St. Louis, MO, USA) in a humidified environment at 37 ℃ and 5% CO_2_. Cells used for experiments were in the logarithmic growth phase.

Total RNA was extracted from cells using the RNA pure kit from Tiangen Biotech Co. (Beijing, China), which was reverse transcribed into cDNA using a reverse transcription kit (Takara, Dalian, China). Next, qRT‐PCR was performed using the FastStart Universal SYBR Green Master (Roche, Basel, Switzerland) on an ABI PRISM 7300plus Sequence Detection system (Applied Biosystems, Foster City, CA, USA). The results were calculated using the $2^{-\Delta \Delta \text{Ct}}$ method, where ΔΔCt = (Ct_RNA_ − Ct_β‐actin_) tumor − (Ct_RNA_ − Ct_β‐actin_) nontumor, and fold change = 2^‐ΔΔCt^. The qRT‐PCR reactions were repeated in triplicate. Primers for qRT‐PCR were synthesized by Sangon Biotech (Shanghai, China), and the sequences may be found in Table 1.

**Table 1:** Real‐time quantitative PCR primer sequences used in this study

Primer name	Forward (5′‐3′)	Reverse (5′‐3′)
β‐actin	CTGGAACGGTGAAGGTGACA	AAGGGACTTCCTGTAACAATGCA
HULC	AGGATACAGCAAGGCCCCAA	GTCCACGATCAGAGTTCCTGC
RP11‐314B1.2	TGGAGGAGACCAGGGTTCAC	GGCTACAATGCCACTGGTCC
RP11‐999E24.3	GTTCACGGTCCTCGGCATTG	AAGCGCTGCATTCCACAAGT
LINC00106	AGTGGTCACCTGAGATGGAGCAG	CGTCTGTCTTACGGCACGAAGC
NR2F1‐AS1	GGACTCGTGCTCCAGATGTTGC	CACTGCCACCGCCATTCATCC
AC018647.3	GAGGCTGACACCGCTATTGGATG	TGTGGAGTTATTGGTGGTGGCTTC
RP5‐1074L1.4	CTGCCTACACCTGCAAGAACTGAG	CTTCTGACTCCAGCAGCAACTACC
PVT1	TTGCTTCTCCTGTTGCTGCTAGTG	TCCTCAGCCTCCAAGCGTTCC
MAGI2‐AS3	CCGCTGCTCTCACCTTGCTTG	TGGTGTCGGAGGAGCTGCTG
MIR99AHG	CGTCTACCTTACTGGCATCGTCTC	GCTCACTAGCAGGCATGGTTGG
RP11‐7K24.3	CTGAGGCAGGCGAATCACTTGAG	GTGATCTCGGCTGACTGCAACC

### 2.9 Statistical analysis

R Studio (R Version 3.4.3), GraphPad Prism 6.0 software (GraphPad Software, San Diego, CA, USA), and PASS 15.0 software were used for the statistical analysis. The log‐rank test was used in the Kaplan‐Meier survival curve analysis, and the ANOVA was used in qRT‐PCR analysis. Any *P*‐value <.05 was considered statistically significant.

## 3 RESULTS

### 3.1 Clinical and demographic patient data

The clinical and demographic information of the 372 gastric cancer patients are shown in Table 2 (Additional File 1). The patients were pathologically diagnosed as having gastric cancer. The median age was 66 years (range: 35‐90 years). The number of male patients was higher than females with a male to female ratio of 1.8. *H pylori* infections were detected in 4.6% of the patients.

**Table 2:** Clinical and demographic data from the 372 patients with gastric cancer

Parameter	Subtype	Patients (%)
Age(years)	>66	194 (52.1%)
	≤66	174 (46.8%)
	Unknown	4 (1.1%)
Gender	Male	239 (64.2%)
	Female	133 (35.8%)
Race	White	237 (63.7%)
	Asian	73 (19.6%)
	Black OR African American	11 (3.0%)
	Unknown	51 (13.7%)
Pathologic stage	Stage Ⅰ	53 (14.2%)
	Stage Ⅱ	114 (30.7%)
	Stage Ⅲ	165 (44.4%)
	Stage Ⅳ	25 (6.7%)
	Unknown	15 (4.0%)
Pathologic T	T1	19 (5.1%)
	T2	79 (21.2%)
	T3	166 (44.6%)
	T4	100 (26.9%)
	TX	8 (2.2%)
Pathologic N	N0	110 (29.6%)
	N1	96 (25.8%)
	N2	75 (20.2%)
	N3	73 (19.6%)
	NX	18 (4.8%)
Pathologic M	M0	327 (87.9%)
	M1	25 (6.7%)
	MX	20 (5.4%)
*H pylori* infection	Yes	17 (4.6%)
	No	143 (38.4%)
	Unknown	212 (57.0%)

### 3.2 Differentially expressed lncRNAs, miRNAs, and mRNAs

**Figure 2:**
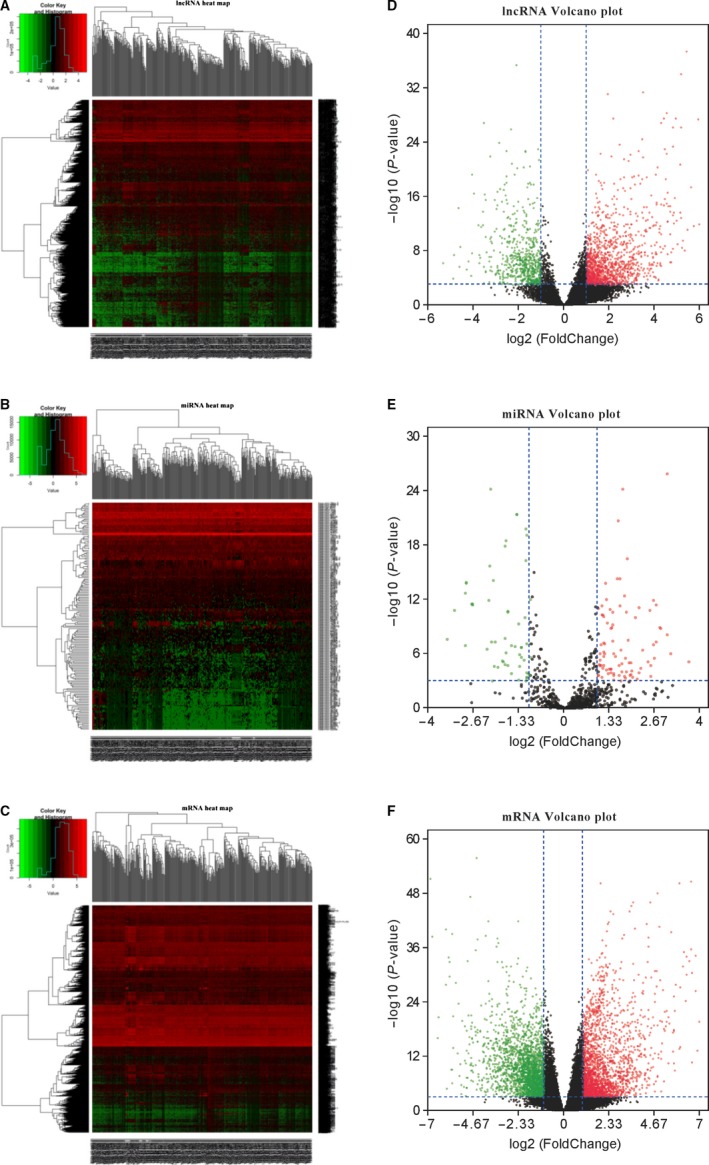
Hierarchical heatmaps and volcano plots presenting for differentially expressed lncRNAs, miRNAs, and mRNAs. Left panels, heat maps for all differentially expressed lncRNAs (A), miRNAs (B), and mRNAs (C) in gastric cancer; Right panels, volcano plots showing lncRNAs (D), miRNAs (E), and mRNAs (F) with fold change ≥ 2 (*P* < .001). Green, downregulated; red, upregulated; black, not differential expressed. lncRNA: long noncoding RNA; miRNA: microRNA

**Figure 3:**
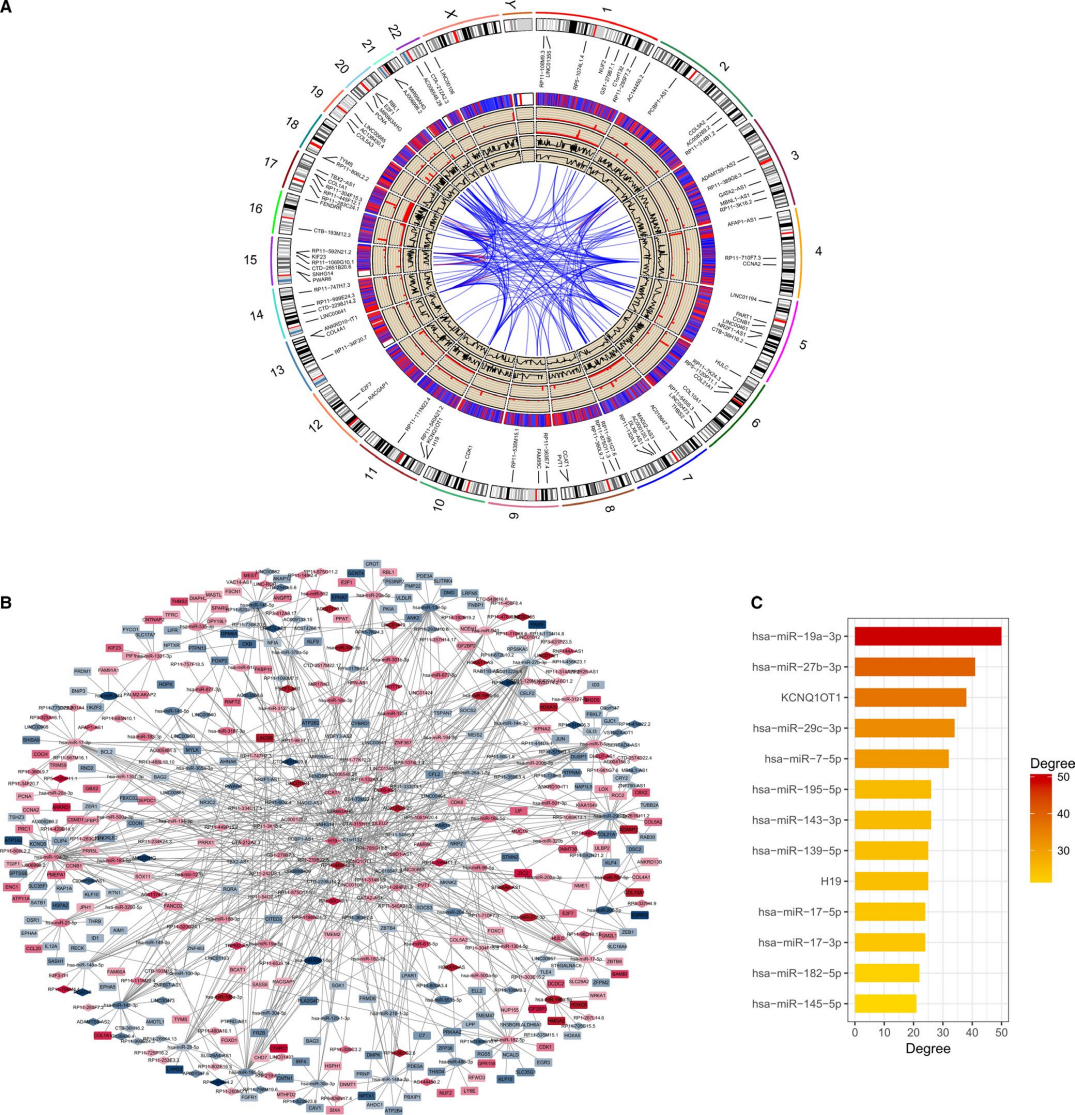
Circos plot representing the lncRNAs and mRNAs on the human chromosomes (A). From outside in, the first layer is the human genome (hg38) chromosome map. The second layer shows the ceRNA subnetwork related to the lncRNAs and mRNAs. The third layer represents the DElncRNAs and DEmRNAs by red and blue bars, respectively. The mean expression values of the DElncRNAs and DEmRNAs are represented in the fourth and fifth circles with the respective fold changes in the sixth and seventh layers (fold change ≥ 2, *P* < .001). The network in the center of the Circos plot represents the relationship of lncRNA or mRNA transcripts involved in the ceRNA subnetwork on the chromosomal location. The red lines indicate the linked RNAs in the same chromosome, while blue is from different chromosomes. Construction of a lncRNA‐miRNA‐mRNA ceRNA network for gastric cancer (B). In the ceRNA network, the blue and red nodes show decreased and increased expression of RNAs, respectively, while the colors are related to the absolute value of fold change. Diamonds represent lncRNAs, ellipses represent miRNAs, rectangles represent mRNAs, and gray edges represent interactions among the lncRNAs‐miRNAs and mRNAs. The bars show the number of node degrees (>20) in the ceRNA network (C). ceRNA: competing endogenous RNA; DElncRNAs: differentially expressed lncRNAs; DEmiRNAs: differentially expressed miRNAs; DEmRNAs: differentially expressed mRNAs

A total of 2221 lncRNAs, 168 miRNAs, and 3879 mRNAs were identified as being differentially expressed between gastric tumor tissues and nontumor tissues with considering |log_2_ fold change| > 1 and an adjusted FDR of *P* < .001. Among them, 1513 lncRNAs, 109 miRNAs, and 1738 mRNAs were upregulated, while 708 lncRNAs, 59 miRNAs, and 2141 mRNAs were downregulated. In addition, 22 lncRNAs, 7 miRNAs, and 46 mRNAs were upregulated more than 10‐fold in the gastric cancer tissues compared to the noncancerous control tissues. The differences in the expression of lncRNAs, miRNAs, and mRNAs among the samples were shown by a hierarchical clustering analysis (Figure 2; Additional File 2). Moreover, the DElncRNAs and DEmRNAs were found to be broadly located in all chromosomes, including chromosomes X and Y (Figure 3; Additional File 2).

### 3.3 Construction of the miRNA‐lncRNA‐mRNA ceRNA network

The lncRNAs and mRNAs targeted by miRNAs were screened basing on the interactions among the DElncRNAs, DEmRNAs, and DEmiRNAs described above. Only 73 of 168 DEmiRNAs were predicted to target 207 of the 2221 DElncRNAs based on DIANA‐LncBase Version 2 experimental module. Next, the 73 selected DEmiRNAs were used to predict the targeted mRNAs using TargetScan, miRDB, and DIANA‐TarBase Version 8. The predicted mRNAs were compared with 3879 specific DEmRNAs, and only 224 mRNAs that existed in both groups were found to be associated with 73 miRNAs. The representative interactions among lncRNAs, miRNAs, and mRNAs are shown in Tables 3 and 4.

**Table 3:** Representative interactions between the miRNAs and lncRNAs for gastric cancer

miRNA	lncRNA
hsa‐miR‐19a‐3p	AC008269.2, AJ006998.2, ANKRD10‐IT1, C1orf132, CCAT1, CTD‐2298J14.2, H19, KCNQ1OT1, LINC00461, MAGI2‐AS3, PART1, PWAR6, RP11‐132A1.4, RP11‐363E7.4, RP11‐429B14.1, RP11‐806L2.2, SNHG14, TBX2‐AS1
hsa‐miR‐27b‐3p	AC010226.4, AC018647.3, C1orf132, CTA‐315H11.2, DLX6‐AS1, KCNQ1OT1, LINC00641, LINC00665, LINC01012, LINC01021, LINC01355, MAGI2‐AS3, MIR663AHG, MIR99AHG, PART1, PVT1, RAB11B‐AS1, RNF144A‐AS1, RP11‐1134I14.8, RP11‐129M16.4, RP11‐16C1.2, RP11‐206M11.7, RP11‐314A20.2, RP11‐314B1.2, RP11‐322D14.2, RP11‐333I13.1, RP11‐363E7.4, RP11‐389G6.3, RP11‐456K23.1, RP11‐540A21.2, RP1‐239B22.5, RP3‐431P23.5, RP4‐785G19.5, RP5‐1061H20.4, RP5‐1074L1.4, SNHG14, VPS9D1‐AS1
hsa‐miR‐7‐5p	AC004158.3, AC012531.25, C1orf132, C9orf147, CTD‐2574D22.4, DLEU2, DLEU7‐AS1, DLX6‐AS1, GS1‐72M22.1, KCNQ1OT1, LINC00641, MAGI2‐AS3, PART1, PWAR6, RP11‐138H8.6, RP11‐16C1.2, RP11‐319G6.3, RP11‐363E7.4, RP11‐418I22.2, RP11‐444D3.1, RP11‐575H3.1, RP11‐592N21.2, RP11‐981G7.6, RP1‐239B22.5, SERTAD4‐AS1, SNHG14, UG0898H09, VSTM2A‐OT1
hsa‐miR‐195‐5p	AC018647.3, C1orf132, DLEU2, DLX6‐AS1, FAM95C, KCNQ1OT1, LINC00473, LINC00641, LINC01355, LINC01433, NR2F1‐AS1, PART1, PTPRD‐AS1, PWAR6, RNF219‐AS1, RP11‐260M2.1, RP11‐314B1.2, , RP11‐333I13.1, RP11‐414J4.2, RP11‐6O2.4, RP11‐747H7.3, RP11‐798M19.6, RP11‐802E16.3, SLC26A4‐AS1, SNHG14
hsa‐miR‐143‐3p	AC000120.7, AC018647.3, AC138430.4, ADAMTS9‐AS2, CTB‐193M12.3, CTB‐36H16.2, DLX6‐AS1, H19, KCNQ1OT1, LINC00473, LINC00641, MAGI2‐AS3, NR2F1‐AS1, PART1, PCBP1‐AS1, PWAR6, RP11‐111M22.4, RP11‐285F7.2, RP11‐334C17.5, RP11‐3K16.2, RP11‐999E24.3, SNHG14, ZNF667‐AS1

**Table 4:** Representative interactions between the miRNAs and mRNAs for gastric cancer

miRNA	mRNA
hsa‐miR‐19a‐3p	ATP11A, ATP1A2, ATP2B2, CCNA2, CLIP4, CSMD1, ENC1, FBXO32, FOXP2, HSPA2, IGFBP3, KCNQ5, KLF10, LPP, NCALD, NR3C2, PMEPA1, PRC1, PRICKLE2, PRR5L, RAP1A, RORA, RTN1, SGK1, SLC35F1, SOCS3, SPTSSB, TGIF1, TSHZ3, ZBTB4, ZER1, ZNF367
hsa‐miR‐29c‐3p	ADAM12, CBX2, CCNA2, CDK6, COL21A1, COL4A1, COL5A2, DNMT3B, DSC2, KIAA1549, KLF4, LOX, RAB30, RCC2, SGK1, TUBB2A, ULBP2
hsa‐miR‐182‐5p	ALDH6A1, CDK1, CDK6, CITED2, EGR3, ELL2, GPR158, KLF15, LPP, NCALD, PRKAA2, RECK, RGS5, SH3BGRL, SLC35G1, TMEM47, VLDLR
hsa‐miR‐20a‐5p	CFL2, CROT, CYBRD1, E2F1, EPHA7, GCNT4, PKIA, RBL1, TP53INP2, VLDLR
hsa‐miR‐148a‐3p	AHDC1,ATP2B4, CFL2, COL4A1, DMPK, DNMT1, NPTX1, PBXIP1, PDE5A, PRNP, SIX4

Depending on the interactions between lncRNA‐miRNA and miRNA‐mRNA, we constructed a lncRNA‐miRNA‐mRNA ceRNA network consisting of 207 lncRNAs, 73 miRNAs, and 224 mRNAs with a total of 803 interactions. We found that hsa‐miR‐19a‐3p in the ceRNA network had the highest degree of 50 interactions, which are closely associated with the development and progression of tumors, including gastric cancer.^32^, ^33^ There were many other nodes with more than 20 degrees that likely play roles as hub‐genes in the network (Figure 3B,C). The degree of one node is the number of edges that connect with others in the same network.

### 3.4 Functional analysis of the ceRNA network‐associated DEmRNAs

**Figure 4:**
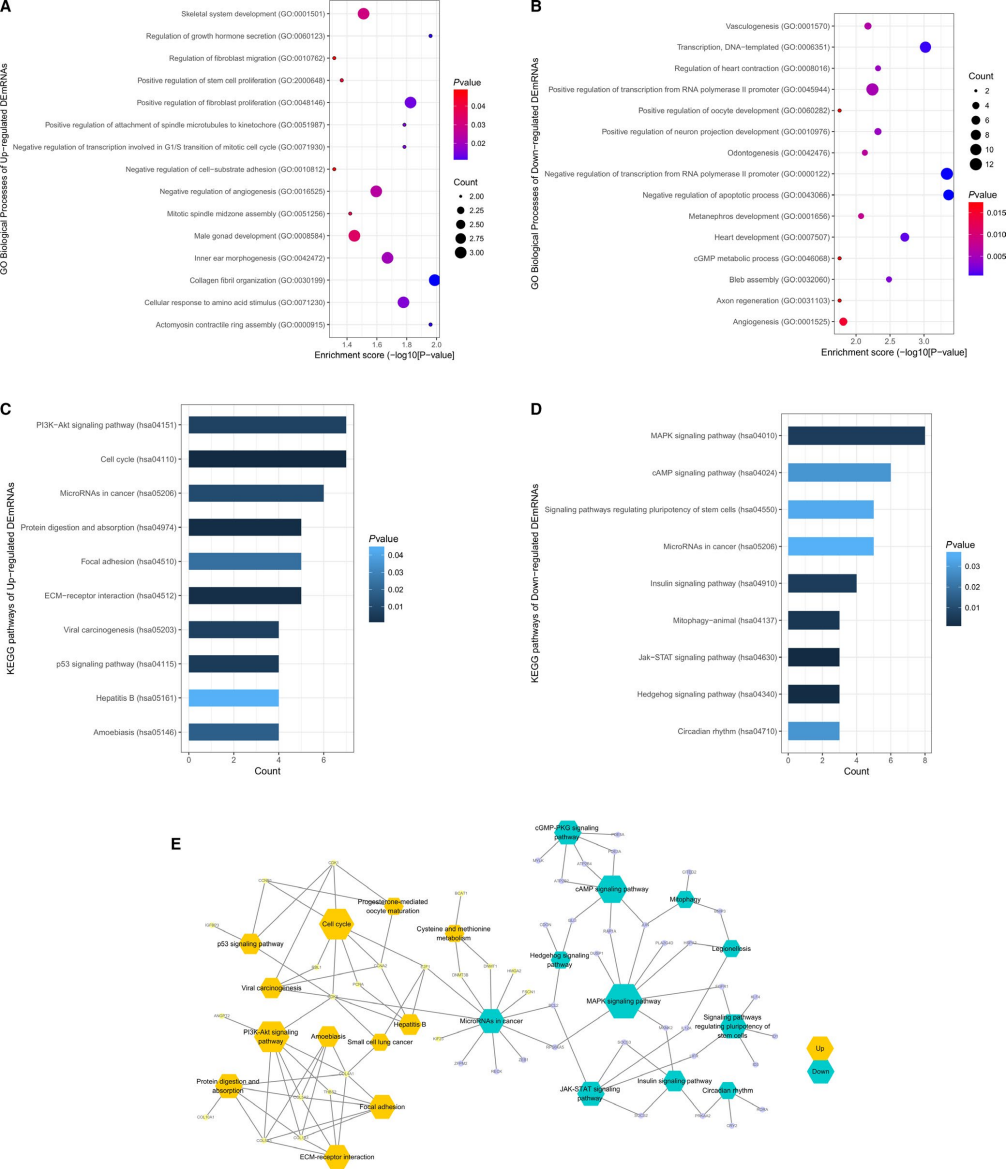
The enrichment analysis of GO and KEGG pathway for ceRNA network‐related DEmRNAs. Top 15 GO biological process terms (*P* < .05) of the upregulated and downregulated ceRNA‐related DEmRNAs, respectively (A, B). Top 10 KEGG pathways (*P* < .05) of the upregulated and downregulated ceRNA‐related DEmRNAs, respectively (C, D). Interactions and overlapping among the important KEGG pathways. Hexagons indicate enriched pathways, while ellipses are the mRNAs (E). Yellow represents upregulated expression, while green is downregulated expression. GO: Gene Ontology; KEGG: Kyoto Encyclopedia of Genes and Genomes

At present, the function of most lncRNAs has not been studied extensively. By analyzing the function of ceRNA network‐associated DEmRNAs, we can predict the role of lncRNAs in gastric cancers. We utilized these DEmRNAs for GO and KEGG pathway analysis using DAVID, which was combined with Metascape bioinformatic tools. The top 15 GO biological process terms and top 10 KEGG pathways of upregulated and downregulated genes, basing on the *p*‐values, were chosen for biological function analysis (Figure 4). Among these pathways, the JAK‐STAT, MAPK, and PI3K‐Akt signaling pathways and ECM‐receptor interactions have been reported to be correlated with the proliferation, invasion, and metastasis of cancer in patients.^34^, ^35^, ^36^, ^37^

### 3.5 Module screening of the protein‐protein interaction (PPI) network

**Figure 5:**
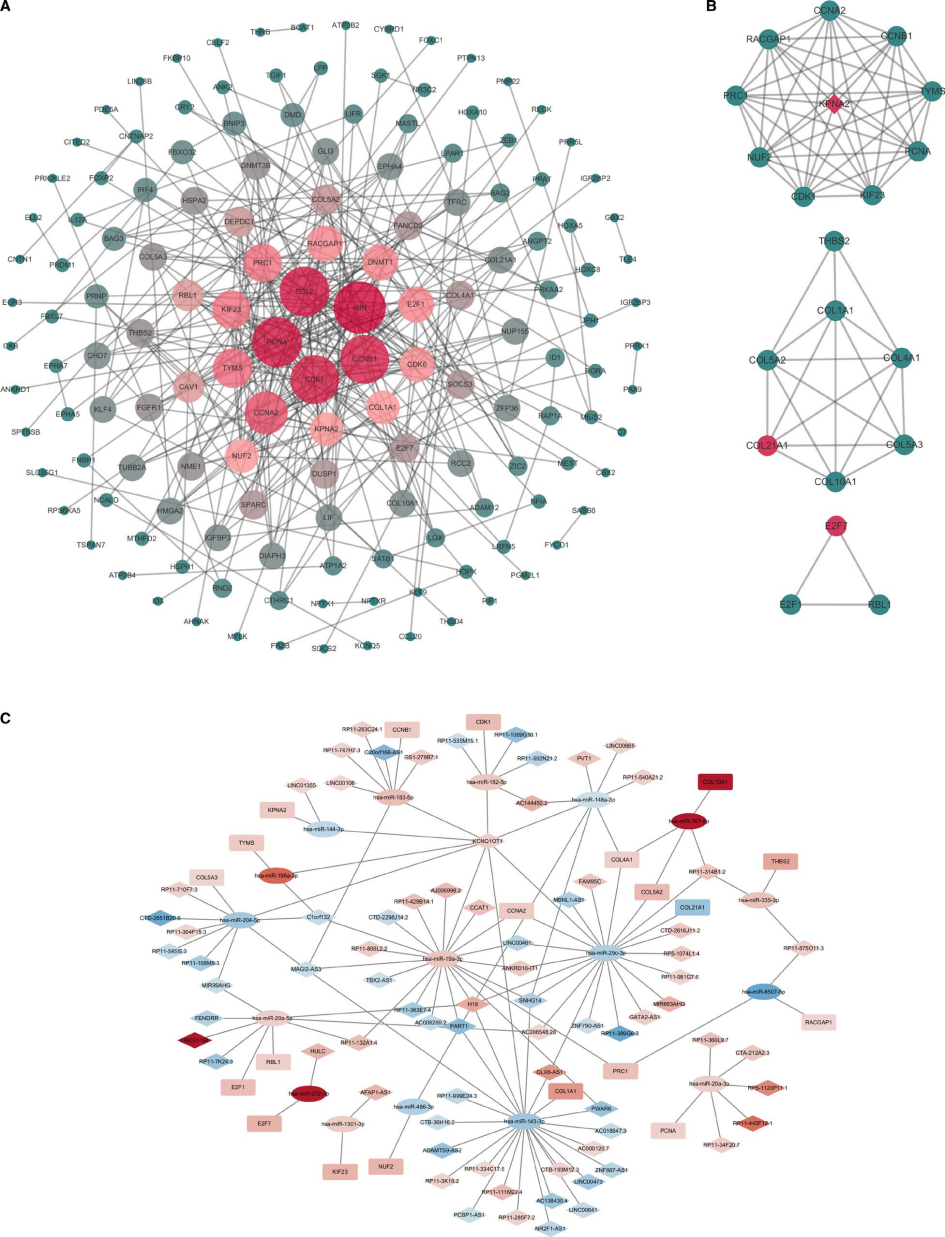
The protein‐protein interaction (PPI) network of ceRNA network‐related DEmRNAs (A). The nodes denote DEmRNAs (confidence score > 0.4) and the size of the nodes represents the degree of each node. Top three modules (module 1‐3) from the PPI network modularized by plug‐in MCODE (B). CeRNA subnetwork based on the modularization. Blue represents downregulated expression, while red represents upregulated expression (C). Diamonds indicate lncRNAs, ellipses show miRNAs, rectangles show mRNAs, and gray edges show interactions among the lncRNA‐miRNA‐mRNA. PPI: protein‐protein interaction; DEmRNAs: differentially expressed mRNAs

Based on the STRING database, we used the ceRNA‐related DEmRNAs to build a PPI network for investigating the function of DEmRNAs at the protein level, along with filtering of functional genes. This PPI network consisted of 145 nodes and 318 edges, and the top 5 predominant nodes with highest degrees were JUN (degree = 27), BCL2 (degree = 25), PCNA (degree = 25), CCNB1 (degree = 24), and CDK1 (degree = 24). Next, we used the plug‐in MCODE to analyze the network and chose the top 3 modules (module 1‐3) for further analysis (Figure 5A,B). The functional analysis of the mRNAs from module 1‐3 showed that these genes were primarily associated with the cell cycle, protein digestion and absorption, PI3K‐Ak signaling, and ECM‐receptor interactions (Table 5).

**Table 5:** GO biological process terms and KEGG enriched pathways for top 3 modules

Term	Genes	P‐value
GO biological process		
Module 1		
(GO:0 000 915) Actomyosin contractile ring assembly	KIF23, RACGAP1	0.0013
(GO:0 051 256) Mitotic spindle midzone assembly	KIF23, RACGAP1	0.0026
(GO:0 000 281) Mitotic cytokinesis	KIF23, RACGAP1	0.0136
(GO:0 032 467) Positive regulation of cytokinesis	KIF23, RACGAP1	0.0142
(GO:0 030 855) Epithelial cell differentiation	CDK1, PCNA	0.0308
Module 2		
(GO:0 071 230) Cellular response to amino acid stimulus	COL4A1, COL1A1, COL5A2	6.14E‐05
(GO:0 030 199) Collagen fibril organization	COL1A1, COL5A2	0.0094
Module 3		
(GO:0 071 930) Negative regulation of transcription involved in G1/S transition of mitotic cell cycle	E2F1, E2F7	7.07E‐04
(GO:0 006 351) Transcription, DNA‐templated	E2F1, E2F7	0.0023
KEGG pathway		
Module 1		
(hsa04110) Cell cycle	CCNB1, CDK1, PCNA, CCNA2	0.0001
(hsa04914) Progesterone‐mediated oocyte maturation	CCNB1, CDK1, CCNA2	0.0023
(hsa04115) p53 signaling pathway	CCNB1, CDK1	0.0564
Module 2		
(hsa04974) Protein digestion and absorption	COL4A1, COL21A1, COL1A1, COL5A3, COL5A2, COL10A1	1.57E‐09
(hsa04512) ECM‐receptor interaction	COL4A1, COL1A1, COL5A3, THBS2, COL5A2	3.79E‐07
(hsa04510) Focal adhesion	COL4A1, COL1A1, COL5A3, THBS2, COL5A2	1.23E‐05
(hsa05146) Amoebiasis	COL4A1, COL1A1, COL5A3, COL5A2	7.28E‐05
(hsa04151) PI3K‐Akt signaling pathway	COL4A1, COL1A1, COL5A3, THBS2, COL5A2	8.45E‐05
(hsa04611) Platelet activation	COL1A1, COL5A3, COL5A2	0.0051
Module 3		
(hsa04110) Cell cycle	E2F1, RBL1	0.0181

### 3.6 Key lncRNAs clinical feature analysis

Based on the functional analysis described above, we used the mRNAs involved in PPI module 1‐3 to reconstruct a ceRNA subnetwork with 76 lncRNAs, 17 miRNAs, and 20 mRNAs (Figure 5C). The 76 lncRNAs involved in the ceRNA subnetwork were selected as key lncRNAs. For further analysis, we identified several correlations between these lncRNAs and clinical signatures, including gender, age, tumor stage, lymphatic metastasis, distant metastasis, and *H pylori* infection. The gastric cancer samples were classified into several subgroups basing on individual clinical features. Next, we performed a comparative analysis of these 76 lncRNAs expression profiles setting the thresholds as |log_2_ fold change| > 1 and an adjusted FDR *P* < .05. highly up‐regulated in liver cancer (HULC) and RP11‐314B1.2 were found to be highly associated with clinical features of the patients, suggesting that they may play roles in the development and progression of gastric cancer (Table 6).

**Table 6:** Correlation between the key lncRNAs of gastric cancer and their clinical features. The lncRNAs were correlated with clinical features if |log_2_ fold change|> 1 and *P* < .05

	Upregulated	Downregulated
Gender (male vs female)		HULC
Age (>66 vs ≤66)	HULC, C20orf166‐AS1	RP11‐314B1.2
Tumor stage (Stage III, IV vs I, II)	HULC, RP11‐314B1.2	LINC00473
Tumor infiltration (T4 T3 vs T2 T1)	HULC, RP11‐314B1.2	
Lymphatic metastasis (yes vs no)	HULC	RP11‐314B1.2, LINC00473
Distant metastasis (yes vs no)	HULC, C20orf166‐AS1, PART1, RP11‐389G6.3, DLX6‐AS1	CTD‐2651B20.6, CCAT1
*H pylori* infection (yes vs no)	HULC, RP11‐445F12.1, MIR663AHG, LINC00473, AC006548.28, GATA2‐AS1, AC138430.4, CTD‐2651B20.6, RP11‐283C24.1	AC144450.2, MBNL1‐AS1

**Figure 6:**
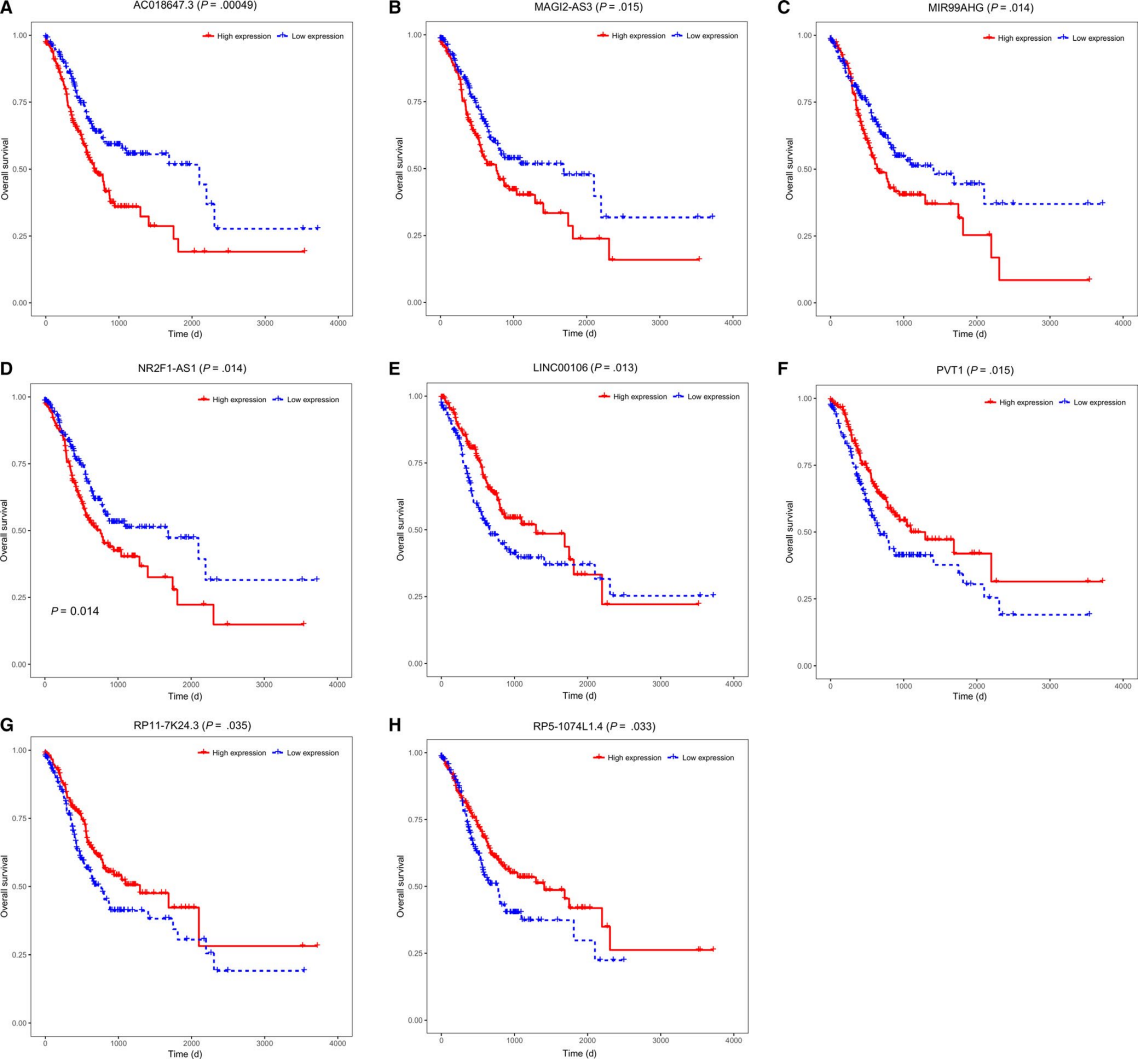
Kaplan‐Meier survival curves of eight lncRNAs (log‐rank *P* < .05). AC018647.3 (A), MAGI2‐AS3 (B), MIR99AHG (C) and NR2F1‐AS1 (D) were negatively associated with overall survival; LINC00106 (E), PVT1 (F), RP11‐7K24.3 (G) and RP5‐1074L1.4 (H) were positively associated with overall survival. The median expression value of each lncRNA was set as the cutoff value for dividing the 372 patients into two groups separately; red lines indicate high expression of lncRNA and blue lines indicate low expression of lncRNA

The overall survival of the 76 key lncRNAs was also analyzed using the Kaplan‐Meier curve analysis. The 372 patients with complete clinical information were divided into high and low expression groups based on the median value of the expression for each lncRNA, and the results showed that eight lncRNAs were associated with the overall survival of patients. Among them, AC018647.3, MAGI2‐AS3, MIR99AHG, and NR2F1‐AS1 were negatively associated with overall survival, whereas LINC00106, PVT1, RP5‐1074L1.4, and RP11‐7K24.3 were positively correlated with overall survival (log‐rank *P* < .05) (Figure 6). These results suggested that LINC00106, PVT1, RP5‐1074L1.4, and RP11‐7K24.3 may play protective roles in the development of gastric cancer.

### 3.7 lncRNAs are associated with overall survival and risk stratification

**Figure 7:**
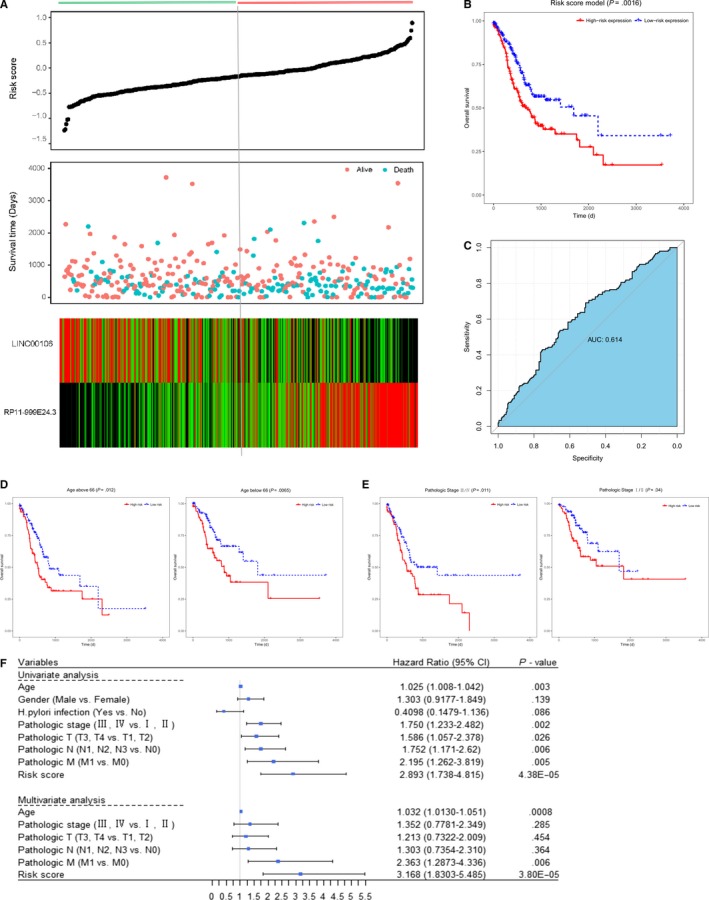
The risk score distribution and survival status of the prediction model, along with the heatmap, of the two prognostic lncRNAs. Vertical line means the median risk factor (−0.23) that divides the patients into low‐risk and high‐risk groups (A). Kaplan‐Meier survival curve shows significant difference between patients with high‐risk and low‐risk scores (log‐rank *P* = .0016) (B). ROC curve (AUC:0.614) of the risk score model (C). Kaplan‐Meier survival curve for elder patients (age > 66, n = 194) and younger patients (age ≤ 66, n = 174) (D). Kaplan‐Meier survival curve for patients with stages III‐IV (n = 187) and stages I‐II (n = 162) (E). The middle point represents the HR, and the length of the line represents the 95% CI for each indicator (F). ROC: receiver operating characteristic; AUC: area under curve; CI: confidence interval; HR: hazard ratio

Univariate Cox regression analysis was performed on the relative expression of 76 key lncRNAs, and identified four lncRNAs that were closely associated with overall survival (*P* < .01), including RP11‐999E24.3 (*P* = .000715), MAGI2‐AS3 (*P* = .00589), LINC00106 (*P* = .00758), and AC018647.3 (*P* = .00793). Then, based on the correlation coefficient of the expression of lncRNAs in the multivariate Cox regression analysis, we selected two lncRNAs to construct a risk factor prediction linear model as follows: risk score = (0.6352 × expression value of RP11‐999E24.3+(−0.5783) × expression value of LINC00106). Then, we performed a Kaplan‐Meier analysis on the patients who were divided into high‐risk and low‐risk groups using the median risk factor as a cutoff point. The result showed significant differences in the overall survival between the two groups (log‐rank *P* < .05), and the mean overall survival days of the high‐risk group (543 days) were shorter than that of the low‐risk group (611.8 days).The area under curve (AUC) in the ROC analysis was 0.614, indicating that the risk score model has high sensitivity and specificity (Figure 7A‐C). All patients were divided by age into elder (age > 66) and younger (age ≤ 66) groups, the result of Kaplan‐Meier analysis showed that the risk score model was a potential prognostic factor in both elder and younger gastric patient groups (elder group, log‐rank *P = *.012; younger group, log‐rank *P = *.0065) (Figure 7D). Then the patients with pathologic stages I‐II and pathologic stages III‐IV were classified into two groups, to evaluate whether the risk score model can predict the overall survival time of gastric patients in different pathologic stage; the results showed that the differences of overall survival time between high‐risk and low‐risk groups were significant in patients of pathologic stages Ⅲ‐Ⅳ and pathologic stages I‐II (stages III‐IV, log‐rank *P = *.011; stages I‐II, log‐rank *P = *.04) (Figure 7E). So we speculated that the prognostic potential of the risk score model may be age‐independent and exists in the development of gastric cancer. Finally, we used univariate Cox regression analysis to analyze the risk score model and other clinical features, and found that the prognostic value of pathologic stage, pathologic T, pathologic N, and pathologic M was statistically significant, similar to risk score model. Through multivariate Cox regression analysis, we found that pathologic stage, pathologic T, and pathologic N were not associated with the prognosis of gastric cancer patients, and the risk score model and pathologic M were independent prognostic indicators of overall survival time for gastric cancer patients (Figure 7F). And, we used the Tests for Two Survival Curves Using Cox's Proportional Hazards Model to perform power calculation. In our study, the number of events is 149 (62 in the low‐risk score group and 87 in the high‐risk score group) and the actual hazard ratio is 3.180. Results showed that, with an overall sample size of 372 subjects (of which 186 are in the low‐risk score and 186 are in the high‐risk score group), the two‐sided test can achieve 100% power at a 0.025 significance level. Above all, we speculated that the two lncRNAs might not only aberrantly express in gastric cancer but may also be closely associated with overall survival of gastric cancer patients.

### 3.8 Correlations between lncRNAs and mRNAs

**Figure 8:**
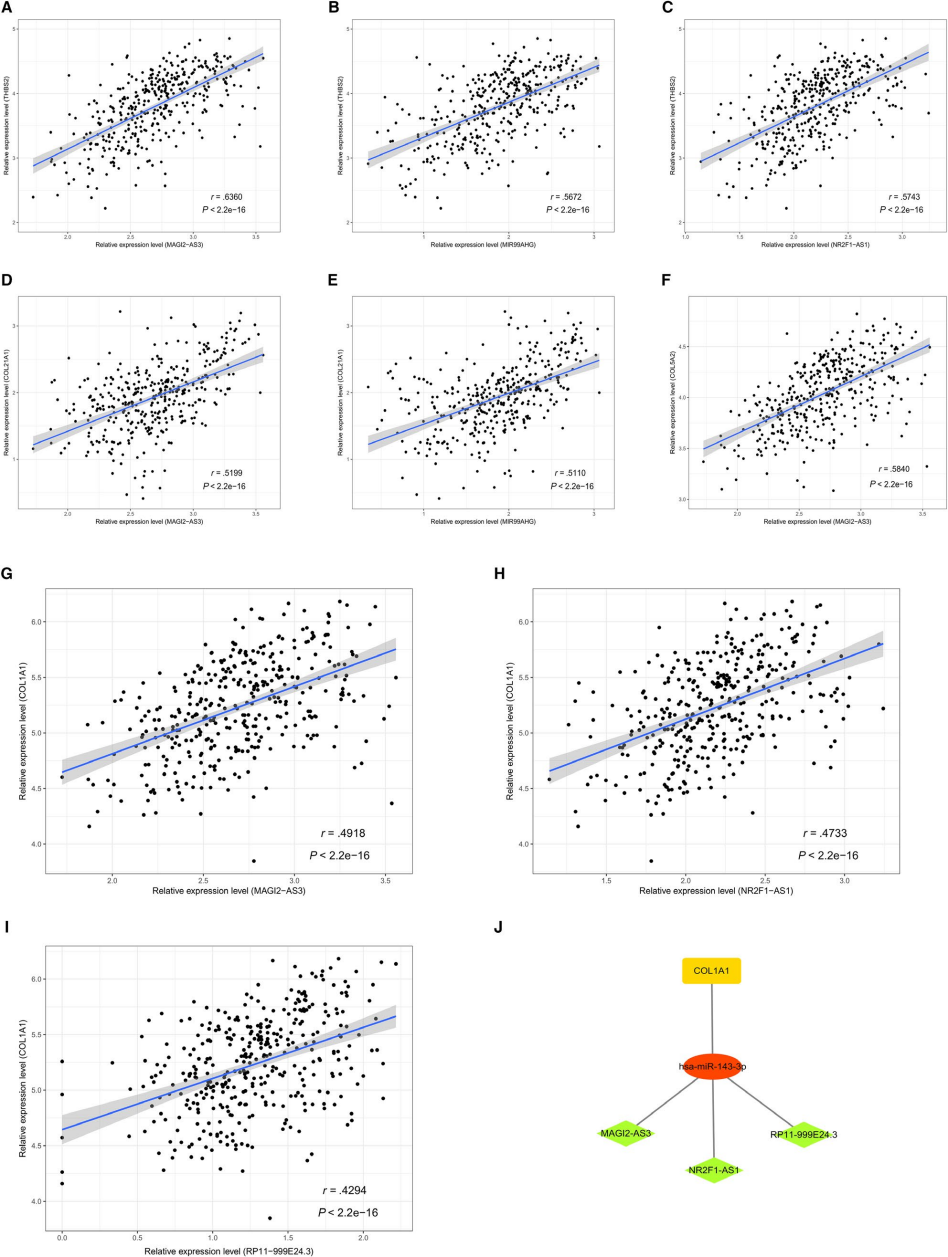
Linear regression analysis between key lncRNAs‐ and ceRNA subnetwork‐related mRNAs (n = 372). The gray area around the blue line represents 95% CI. The represent lncRNA‐mRNA pairs with higher correlations (*r * > 0.3, *P* < .05) included MAGI2‐AS3‐THBS2 (A), MIR99AHG‐THBS2 (B), NR2F1‐AS1‐THBS2 (C), MAGI2‐AS3‐COL21A1 (D), MIR99AHG‐COL21A1 (E), MAGI2‐AS3‐COL5A2 (F), MAGI2‐AS3‐COL1A1 (G), NR2F1‐AS1‐COL1A1 (H) and RP11‐999E24.3‐COL1A1 (I). Identified lncRNA‐miRNA‐mRNA axis are showed as a network map (J). Green diamond: lncRNA; red ellipse: miRNA; yellow rectangle: mRNA. CI: confidence interval

lncRNAs act via various biological mechanisms, not only as ceRNAs to affect the expression of genes but also to regulate the expression of mRNAs by cis‐patterns or distant genes by trans‐patterns.^38^, ^39^ We performed linear regression analysis on the nine overall survival‐related lncRNAs and 20 mRNAs that were involved in the ceRNA subnetwork, and the positive correlations were selected for 34 lncRNA‐mRNA pairs. The results showed that MAGI2‐AS3, NR2F1‐AS1, and MIR99AHG were highly correlated with THBS2, and MAGI2‐AS3‐COL21A1, MAGI2‐AS3‐COL5A2, and MIR99AHG‐COL21A1 also had higher correlations (*r* > .5). Then, we found that miR‐143‐3p is a shared miRNA between lncRNAs and mRNAs, including MAGI2‐AS3‐miR‐143‐3p‐COL1A1, NR2F1‐AS1‐miR‐143‐3pCOL1A1, and RP11‐999E24.3‐miR‐143‐3p‐COL1A1, and speculated that miR‐143‐3p and COL1A1 may be key genes involved in ceRNA pathways for gastric cancer (Figure 8).

### 3.9 Validation of dysregulated lncRNAs in qRT‐PCR

**Figure 9:**
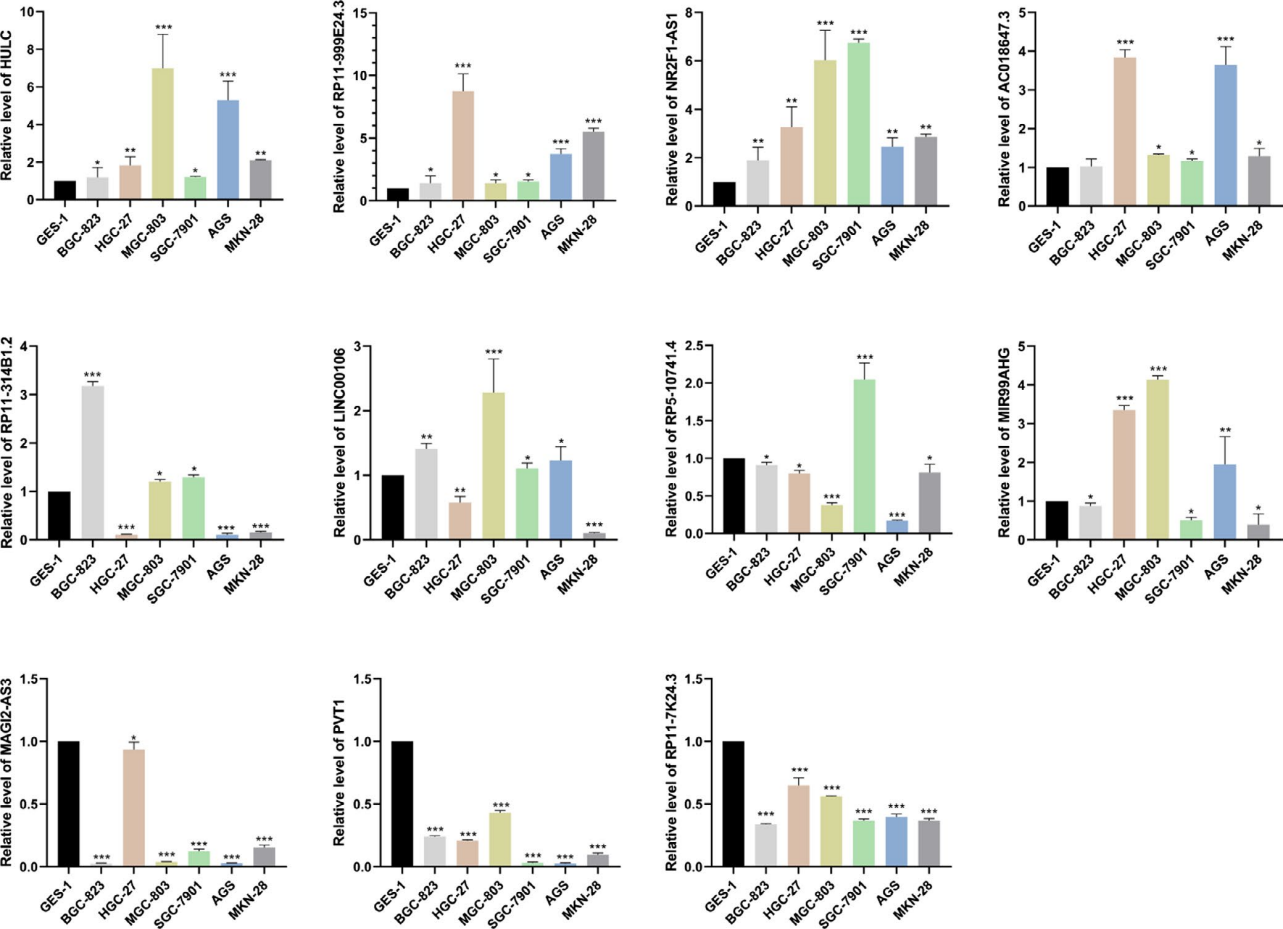
The levels of HULC, RP11‐314B1.2, AC018647.3, MAGI2‐AS3, MIR99AHG, NR2F1‐AS1, LINC00106, PVT1, RP5‐1074L1.4, RP11‐7K24.3, and RP11‐999E24.3 expression in gastric cancer cell lines (BGC‐823, HGC‐27, MGC803, SGC7901, AGS, and MKN28) and the normal noncancerous cell line GES‐1 were detected using qRT‐PCR. Experiments were performed in triplicate, ^*^ *P* < .05, ^**^ *P* < .01, ^***^ *P* < .001 by ANOVA

To validate the bioinformatics analysis results, we utilized qRT‐PCR to detect the expression of specific lncRNAs. Through our confirmation, HULC (related to clinical features) was overexpressed in gastric carcinoma cell lines. In addition, RP11‐314B1.2 was overexpressed in BGC‐823, MGC‐803, and SGC‐7901 cells but was expressed at low levels in HGC‐27, AGS, and MKN28 cells. Moreover, the overall survival‐related lncRNAs RP11‐999E24.3, NR2F1‐AS1, and AC018647.3 were overexpressed in BGC‐823, MGC‐803, HGC‐27, SGC7901, AGS, and MKN28 cells, and MAGI2‐AS3, PVT1, and RP11‐7K24.3 showed low levels of expression in BGC‐823, MGC‐803, HGC‐27, SGC7901, AGS, and MKN28 cells (Figure 9).

## 4 DISCUSSION

Gastric cancer is the fifth most common form of cancer worldwide.^1^, ^2^ Gastroscopy is the most common method for examining gastric cancer, and surgery is the only effective method to effectively treat disease. However, most patients with gastric cancer are asymptomatic for years, resulting in the majority of patients being diagnosed with advanced stage disease. This may also be due to the lack of effective diagnostic biomarkers for the early diagnosis and treatment of gastric cancer. Bioinformatics and other technologies can be used to identify new biomarkers, yet there has been minimal success in this field. Recently, multiple studies uncovered the role of lncRNAs in the development of tumors.^40^, ^41^ According to the ceRNA hypothesis, ceRNA can bind miRNAs through MREs to affect miRNA‐induced gene silencing, and ceRNA includes various types of RNA transcripts, such as circRNAs, lncRNAs, pseudogenes, and protein‐encoding mRNAs.^16^ Studies have reported that miRNAs and their ceRNA targets, including lncRNAs and mRNAs, can build complex regulatory networks that are closely related to the occurrence and progression of many carcinomas.^42^

In this study, we identified 2221 lncRNAs, 168 miRNAs, and 3879 mRNAs that were differentially expressed between gastric tumor tissues and noncancerous tissues based on the RNA expression profiles from the TCGA. Next, we constructed a lncRNA‐miRNA‐mRNA ceRNA network by utilizing the bioinformatic tools and performed GO and KEGG functional analyses on the ceRNA networks‐related DEmRNAs. Through modularization, the core RNAs of each module were chosen as the key genes for further investigation. STRING was used to establish a PPI network for screening the modules to extract the ceRNA subnetwork. lncRNAs in the subnetwork were selected as the key lncRNAs, then we investigated the correlations between these lncRNAs and clinical features of gastric cancer patients. Nine of the 76 key lncRNAs were found to be associated with the overall survival of patients. Moreover, two lncRNAs correlated with overall survival were screened to establish a predictive model. Our findings presented in this study were validated by qRT‐PCR in cells.

Using GO and KEGG, we analyzed the ceRNA networks‐related DEmRNAs. According to ceRNA hypothesis, lncRNAs can regulate the expression of mRNAs by acting as miRNA sponges, suggesting that the biological function of lncRNAs may be similar to that of mRNAs. Through GO annotation, we found that specific genes may be involved in the several biological processes, including the regulation of apoptotic processes, cell cycle, and angiogenesis. The enriched KEGG pathways with upregulated expression of DEmRNAs included the p53 signaling pathway, ECM‐receptor interaction, and PI3K‐Akt signaling pathway. The pathways with downregulated expression of DEmRNAs included the JAK‐STAT signaling pathway and MAPK signaling pathway, which were previously found to be associated with malignant tumors.^34^, ^35^, ^36^, ^37^, ^43^ The PI3K‐Akt signaling pathway has been shown to participate in cell proliferation, migration, differentiation, survival, and trafficking in vitro.^44^

Through our module analysis of the PPI network, the mRNAs in the main modules were used as the core genes to further extract a subnetwork from the entire ceRNA network. Modularization can simplify the complex network into several modules for further research, and cores in the modules may have important functions. Several studies have shown that the core genes of the modules, such as COL1A1 and COL10A, participate in the invasion and metastasis of gastric carcinomas.^45^, ^46^, ^47^ Thrombospondin 2 (THBS2) has also been reported to inhibit tumor growth and angiogenesis and has been recommended as a potential diagnostic biomarker for several types of cancer.^48^, ^49^ In addition, karyopherin subunit alpha 2 (KPNA2) is associated with cell proliferation, migration, and apoptosis, and has been found upregulated in many cancers and is an indicator of a poor prognosis.^50^, ^51^ The abnormal activation of cyclin‐dependent kinase 1 (CDK1) has been reported to be associated with proliferation and apoptosis regulation of cancer cells.^52^ The functional analysis showed that these genes were also involved in the cell cycle, ECM‐receptor interactions, and the PI3K‐Akt signaling pathway. lncRNAs were correlated with the core genes in the modules through the co‐expression network and may play a regulatory role in the biological functions. Thus, the lncRNAs in the ceRNA subnetworks may be chosen as key lncRNAs for the analysis.

In the analysis of the correlation of lncRNAs and clinical features, we found that HULC was most closely associated with the clinical features of patients, including tumor stage, lymphatic and distant metastasis, and *H pylori* infection. Previously, HULC was found to be highly expressed in gastric cancer, which was accompanied by lymph node and distant metastases, and it can enhance autophagy and the EMT phenotype.^53^ These studies also confirmed the reliability of our analysis used in the current study. Moreover, RP11‐314B1.2 was also associated with several clinical features, including age, tumor stage, tumor infiltration, and lymphatic metastasis. However, there are few reports on the correlations of RP11‐314B1.2 with cancers. Thus, the genes related to RP11‐314B1.2 that were predicted by the ceRNA network included five mRNAs (CCNA2, COL21A1, COL5A2, COL4A1, and THBS2) mediated by three miRNAs (hsa‐miR‐29c‐3p, hsa‐miR‐335‐3p, and hsa‐miR‐767‐5p). Through the analysis mentioned above, we suspected that RP11‐314B1.2 might also function in the progression and development of tumors, and we confirmed the differential expression of HULC and RP11‐314B1.2 in gastric cancer cells when compared with noncancerous cells by qRT‐PCR.

The depth of tumor infiltration (pathologic T) is an established independent risk factor for gastric cancer that has been applied to the staging system for medical guidance, and upper gastrointestinal endoscopy is the current gold standard for the diagnosis of gastric cancer.^54^ Recently, several research groups have established new predictive models to more accurately and quickly predict the prognosis of gastric cancer patients.^55^, ^56^ In this study, nine lncRNAs (AC018647.3, MAGI2‐AS3, MIR99AHG, NR2F1‐AS1, LINC00106, PVT1, RP5‐1074L1.4, RP11‐7K24.3, and RP11‐999E24.3) were found to be associated with the overall survival of patients. Among these lncRNAs, PVT1 is one of the best studied lncRNAs. The growing evidence shows that PVT1 promotes proliferation, invasion, metastasis, and drug resistance in many cancer cells,^57^ and can act as a prognostic indicator.^58^ MAGI2‐AS3 is another confirmed lncRNA that can suppress hepatocellular carcinoma cell proliferation and migration by acting as an endogenous sponge of miR‐374b‐5p.^59^ NR2F1‐AS1 can regulate hepatocellular carcinoma oxaliplatin resistance through targeting miR‐363‐ABCC1 pathway.^60^ In our analysis, MAGI2‐AS3 and NR2F1‐AS1 showed highly positive correlation with COL1A1 via miR‐143‐3p. In addition, the risk score model based on linear combination in the Cox regression analysis was identified to be an independent prognostic indicator with high sensitivity and specificity through ROC analysis. Therefore, our ceRNA network identified not only a series of confirmed lncRNAs, but also potential nonconfirmed lncRNAs in gastric cancer, such as RP11‐999E24.3. Above all, we hypothesized that these lncRNAs may be involved in the development of gastric carcinomas and may impact the prognosis of patients with gastric cancer, which has excellent clinical research value.

## 5 CONCLUSIONS

In conclusion, we constructed a ceRNA network by analyzing the lncRNA, miRNA, and mRNA expression profiles of gastric cancers from the TCGA database. We identified one upregulated lncRNA that may be involved in tumorigenesis and nine aberrantly expressed lncRNAs that may be useful to predict the overall survival of patients with gastric cancer. The potential mechanisms of these lncRNAs in gastric cancer should be researched to determine their feasibility as diagnostic or therapeutic biomarkers.

## CONFLICT OF INTEREST

The authors declare no competing interests.

## Data Availability

The data that support the findings of this study are available on request from the corresponding author.
